# Dual Simian Foamy Virus/Human Immunodeficiency Virus Type 1 Infections in Persons from Côte d’Ivoire

**DOI:** 10.1371/journal.pone.0157709

**Published:** 2016-06-16

**Authors:** William M. Switzer, Shaohua Tang, HaoQiang Zheng, Anupama Shankar, Patrick S. Sprinkle, Vickie Sullivan, Timothy C. Granade, Walid Heneine

**Affiliations:** Division of HIV/AIDS Prevention, National Center for HIV/AIDS, Viral Hepatitis, STD, and TB Prevention, Centers for Disease Control and Prevention, Atlanta, GA 30329, United States of America; University of Pittsburgh Center for Vaccine Research, UNITED STATES

## Abstract

Zoonotic transmission of simian retroviruses in West-Central Africa occurring in primate hunters has resulted in pandemic spread of human immunodeficiency viruses (HIVs) and human T-lymphotropic viruses (HTLVs). While simian foamy virus (SFV) and simian T- lymphotropic virus (STLV)-like infection were reported in healthy persons exposed to nonhuman primates (NHPs) in West-Central Africa, less is known about the distribution of these viruses in Western Africa and in hospitalized populations. We serologically screened for SFV and STLV infection using 1,529 specimens collected between 1985 and 1997 from Côte d’Ivoire patients with high HIV prevalence. PCR amplification and analysis of SFV, STLV, and HIV/SIV sequences from PBMCs was used to investigate possible simian origin of infection. We confirmed SFV antibodies in three persons (0.2%), two of whom were HIV-1-infected. SFV polymerase (*pol*) and LTR sequences were detected in PBMC DNA available for one HIV-infected person. Phylogenetic comparisons with new SFV sequences from African guenons showed infection likely originated from a *Chlorocebus sabaeus* monkey endemic to Côte d’Ivoire. 4.6% of persons were HTLV seropositive and PCR testing of PBMCs from 15 HTLV seroreactive persons identified nine with HTLV-1 and one with HTLV-2 LTR sequences. Phylogenetic analysis showed that two persons had STLV-1-like infections, seven were HTLV-1, and one was an HTLV-2 infection. 310/858 (53%), 8/858 (0.93%), and 18/858 (2.1%) were HIV-1, HIV-2, and HIV-positive but undifferentiated by serology, respectively. No SIV sequences were found in persons with HIV-2 antibodies (n = 1) or with undifferentiated HIV results (n = 7). We document SFV, STLV-1-like, and dual SFV/HIV infection in Côte d’Ivoire expanding the geographic range for zoonotic simian retrovirus transmission to West Africa. These findings highlight the need to define the public health consequences of these infections. Studying dual HIV-1/SFV infections in immunocompromised populations may provide a new opportunity to better understand SFV pathogenicity and transmissibility in humans.

## Introduction

Hunting and butchering of wild animals provides nutrition and economic sustenance in many countries across Africa. In Central and West Africa alone, approximately 1 million metric tons of bushmeat are killed every year, of which almost 15% comes from nonhuman primates (NHPs) (www.bushmeat.org). In addition to hunting and consumption by locals, there is also increasing international trade of bushmeat. These socioeconomic and behavioral factors result in frequent and possibly dangerous exposures to NHPs with concomitant increased risks of infection with viral agents, including simian retroviruses [1–5]. 75% of all emerging infectious diseases are estimated to have a zoonotic origin, the majority (37%) of which are RNA viruses [1, 2]. Such historical and contemporaneous hunting of NHPs has resulted in human infection with simian retroviruses, some of which resulted in pandemics, raising concerns of additional primate retrovirus spread to humans. For example, human immunodeficiency viruses types 1 and 2 (HIV-1 and HIV-2, respectively) are recent examples of pandemic retroviruses which originated from cross-species infections from simian immunodeficiency virus (SIV)-infected chimpanzees (*Pan troglodytes*) and gorillas (*Gorilla gorilla*), and sooty mangabeys (*Cercocebus atys*) in West-Central Africa and West Africa, respectively [6]. Human T-lymphotropic viruses types 1–4 (HTLV-1, -2, -3, and -4) have both ancient and contemporaneous cross-species origins from multiple simian T-lymphotropic viruses (STLVs) [7, 8]. HTLV and STLV are collectively referred to as primate T-lymphotropic viruses (PTLVs).

Current evidence shows that simian foamy viruses (SFV), although ancient retroviruses that have co-speciated with their hosts, have more recently jumped species into humans exposed to captive NHPs in North America and Europe or to wild and commensal NHPs in Central Africa (Cameroon, Gabon, Democratic Republic of Congo) or Asia [9–13]. While SFV is an ancient retrovirus that has co-speciated with its primate hosts, most SFV human infections have been epidemiologically and phylogenetically linked to NHP species the persons were exposed to [14]. A human-specific SFV variant has not yet been identified [14]. In contrast to HIV and HTLV, which have known pathogenic consequences and are transmitted from person-to-person, less is known about health outcomes and secondary transmission of SFV infections, which is limited to follow-up of small numbers of apparently healthy infected people for only short periods [14]. Likewise, the lack of systematic studies have limited conclusions about the pathogenicity of SFV in NHPs but which appears to be benign. In contrast, SIV can cause AIDS-like illness in some NHP species naturally and experimentally infected with SIV [15–17] and STLV-1 can cause lymphoma and leukemia in infected NHPs [18].

Little is also known about the prevalence of SFV and other zoonotic retroviruses outside of Central Africa and in sick populations [14]. In this study we assessed the prevalence of simian-origin retroviruses in Côte d’Ivoire, West Africa. We screened specimens collected in the mid and late 1980s and 1990s from a large population of hospital patients and pregnant women for evidence of SFV, SIV- and STLV-like viruses. Besides Nigeria, Côte d’Ivoire has the highest primate diversity in West Africa consisting of 22 taxa of apes and monkeys, including chimpanzee (*Pan troglodytes verus*), baboon (*Papio anubis*), colobines (*Procolobus* and *Colobus* species), sooty mangabeys, (African green monkey (*Chlorocebus sabaeus*), and guenons (*Cercopithecus* species) [19]. Despite a national ban on primate hunting throughout Côte d’Ivoire, about 250 tons of primate bushmeat were extracted from the forests in 1999 for consumption, with overrepresentation of larger species, including sooty mangabeys, red colobus (*P*. *badius*), and the western black-and-white colobus (*C*. *polykomos*) [20]. These findings demonstrate the likelihood of increased risk for exposure to viruses present in these animals which has been confirmed in two recent reports. Firstly, a new HIV-2 lineage from *C*. *atys* in Côte d’Ivoire with highest sequence identity to SIV was identified in an eight year old boy living in a village bordering the Tai National Forest (TNF) [21] who reported consumption of primate bushmeat. The second report from the same group showed evidence for STLV-1-like infection in four persons from this same population likely originating from *C*. *atys* (sooty mangabeys), red colobus monkeys (*Piliocolobus badius badius*) or chimpanzees (*Pan troglodytes verus*) endemic to TNF [22]. Combined, these results detail retrovirus infection of simian origin in Côte d’Ivoire and provide support for the current study.

## Materials and Methods

### Study population and specimen preparation

Whole blood specimens were collected in 1985–1988 (n = 452), 1987–1988 (n = 670) and 1995–1996 (n = 417) from sick patients, pregnant women and newly diagnosed tuberculosis patients in various hospitals in Abidjan, Côte d’Ivoire as part of different HIV studies with Project RETRO-CI [23–27]. Specimens were processed locally for plasma and cryopreserved peripheral blood mononuclear cells (PBMCs), aliquoted, shipped to the CDC on liquid nitrogen (LN2) and archived in LN2. The demographic, clinical and epidemiologic information originally collected for these studies was lost during the multiple custodial transfers and long term storage of the specimens. The original studies were approved by the ethical committee of the Côte d'Ivoire Ministry of Health and the Institutional Review Board of the Centers for Disease Control and Prevention (CDC), Atlanta, Georgia, USA. Informed verbal consent was obtained from all participants. A non-research determination was approved for retroviral testing of these anonymized samples at CDC. 1,539 archived plasma with limited associated PBMC specimens were available for the current study.

### NHP specimen preparation and species inference

Limited information is available on the genetic diversity of SFV in the four *Chlorocebus* species for a more precise phylogenetic comparison with the SFV found in one patient. However, SFVs appear to codiverge with the NHPs [11] allowing for accurate species identification of persons infected with these SFVs [12, 14, 28]. Thus, specimens from a number of each of the *Chlorocebus* species were obtained to obtain and include their SFV sequences in the phylogenetic analyses. Cryopreserved PBMC specimens and PBMC pellets from *Chl*. *tantalus* monkeys from Uganda and the Central African Republic (CAR) were kindly provided by Vanessa M. Hirsch at NIH and Michaela C. Müller-Trutwin at Institut Pasteur, respectively. PBMCs and PBMC DNA from *Chl*. *sabaeus* monkeys were kind gifts of Vanessa Hirsch, Jon Allan at the Southwest Foundation, and Heather Gonsoulin at the New Iberia Research Center. Jon Allan and Francois Villinger at Emory University provided PBMC DNA from *Chl*. *pygerythrus* from Kenya. Cheick Coulibaly at the Paul Ehrlich Institute kindly provided blood specimens from captive *Chl*. *aethiops*. DNA lysates were prepared as described in detail elsewhere [29, 30]. All specimens were collected for other studies or were collected opportunistically and all the previous studies and specimen collections received appropriate animal usage approvals at the respective institutions. DNA integrity was confirmed by PCR of ß-actin sequences [29]. Species identification was recorded in the field and at the local institutions where captive animals were housed and was confirmed on all NHP samples by phylogenetic analysis of mitochondrial cytochrome oxidase subunit II (COX2) sequences as previously described [14]. Neighbor joining trees were constructed in MEGA v6.0.6 for determining the genetic relationships of the COX2 sequences.

### Serologic testing

Plasmas were screened for SFV antibodies using an EIA assay that employs recombinant SFV proteins prepared from complete SFV *gag* genes that were PCR-amplified from canine thymocyte (Cf2Th) cultures infected with either African green monkey (SFVHu1) or chimpanzee (SFVHu6) SFVs previously isolated from humans [28]. Inclusion of viral proteins from both SFVHu1 and SFVHu6 in the serological assays ensures detection of broad seroreactivity to monkey and ape SFV, respectively [31]. Plasmas were tested at 1:100 plasma dilution and each run included SFVcpz and SFVagm-positive control plasmas and two pedigreed SFV-negative human control plasmas. Previously, we have shown that this EIA is 96% sensitive and 97% specific for SFV antibodies using a large number of human and simian plasma from PCR-positive (n = 101) and PCR-negative (n = 605) individuals [31]. Seroreactivity was defined as those samples with an optical density (OD) greater than the cutoff of the mean OD values of the two negative controls plus 0.312, which is three standard deviations of the mean of the observed seroreactivity of the PCR-negative samples. Specimens reactive in the EIA were then tested using a WB assay to confirm the observed seroreactivity as described previously [31]. Samples reactive to p68 and p72 or p70 and p74 Gag doublet proteins, while showing an absence of a similar reactivity to the uninfected CF2Th control antigen, were considered WB-positive and infected with SFV.

Serologic testing for PTLV was performed by using an HTLV-1/2 EIA (HTLV-I/II ELISA v4.0, MP Biomedicals, Solon, OH) followed by confirmation with an HTLV-1-based western blot (WB; HTLV Blot v2.4, MP Biomedicals, Singapore) assay that also includes type-specific antigens that differentiate between HTLV-1 and HTLV-2. This combination of EIA and WB tests has been used successfully to detect PTLV-1, PTLV-2, PTLV-3, and PTLV-4 in humans and in NHPs [8, 29, 32, 33].

A subset (n = 858) of plasma samples were randomly selected and tested for HIV antibodies to estimate the prevalence of HIV-1 and HIV-2 in the study population since all records of the original testing were no longer available. Samples were screened using the BioRad GS HIV-1/HIV-2 *PLUS O* EIA (BioRad Laboratories, Inc., Redmond, CA) with seroreactive samples tested further using the BioRad Multispot HIV-1/HIV-2 Rapid test (BioRad Laboratories, Inc., Redmond, CA) which contains HIV-type specific recombinant envelope proteins and synthetic peptides to differentiate the seroreactivity to HIV-1 from HIV-2.

### SFV and PTLV PCR, proviral load, and sequence analysis

DNA was extracted from cryopreserved PBMCs available from persons with seropositive WB results using the Flexigene DNA extraction kit (Qiagen) and were quantified using a Nanodrop 1000 instrument (Thermo Scientific). DNA integrity was verified using β-actin PCR as described elsewhere [34, 35]. One ug of DNA was input for a generic SFV nested PCR assay to detect 465-bp polymerase (*pol*) and 300-330-bp long terminal repeat (LTR) sequences as described elsewhere [31, 36]. The *pol* and LTR primers can detect highly divergent SFV from a variety of primate species [12, 14, 31]. DNA from tissue culture cells infected with a macaque SFV (SFVmac) was used as a positive control for both PCRs.

Limited information exists regarding viral loads in SFV-infected humans and NHPs [9], which has been associated with increased pathogenicity and transmissibility of other human retroviruses. To investigate SFV proviral loads in PCR-positive persons we recently described a generic quantitative PCR test (qPCR) that detects *pol* sequences using primers SIF4O and SIR1N and the two probes SIP4O and SIP5RON [12]. One μg of DNA, equivalent to about 10^5^ lymphocytes, from SFV PCR-positive persons was tested using the AmpliTaq Gold system (Applied Biosystems/Roche, Branchtown, NJ) on a BioRad CFX96 iCycler (Hercules, CA) for 95°C for 10 min, followed by 55 cycles of 95°C and 52°C for 15 sec each, and 62°C for 30 sec. This qPCR assay was shown to be 100% specific and was able to detect 1–5 input copies of a broad variety of SFV variants from monkeys and apes [12].

Broad detection of PTLV was accomplished using 0.5 ug uncultured PBMC DNA and degenerate *tax* primers PH1FN (5’-YYI TCA GCC CAY TTY CCA GG-3’) and AV46 (5’-KGG RGA IAG YTG GTA KAG GTA-3’) for the first round PCR using the Expand High Fidelity Taq polymerase (Roche). Nested PCR used primers PH2FN (5’- YCC AGG ITT YGG RCA RAG YCT YCT-3’) and AV43 (5’-CCA SRL GGT GTA IAI GTT TTG G-3’) and AmpliTaq enzyme (Life Technologies). Positive controls included DNA from the PTLV-infected cell lines HTLV-1(MT2), HTLV-2(MoT) and STLV-3(PH969) that had consistent detection limits of one infected cell per reaction (0.01 ng/reaction). Further PTLV-1 and -2 resolution was accomplished by PCR amplification of overlapping regions of the 5’ and 3’ HTLV-1 LTR (611-bp) or nearly complete HTLV-2 LTR sequences using primers and conditions reported previously [32, 37].

We designed and validated a new qPCR assay that uses a single set of generic primers and probes in the highly conserved *tax* gene to detect PTLV-1, PTLV-2, PTLV-3, and PTLV-4. 0.5 ug of DNA was tested using the forward 5’-CTG GGA CCC CAT CGA TGG A-3’ and reverse 5’-GGG GTR AGR ACY TTG AGG GT-3’ primers with a 5’-FAM-TCK YTG GGT GGG GAA GGA GGG GAG-BHQ1-3’ probe. Cycling conditions were 95°C for 30 sec, 50°C for 30 sec and 62°C for 30 sec using a BioRad CFX96 iCycler and AmpliTaq Gold enzyme (Life Technologies). To ensure broad reactivity of the assay for both detecting and quantification of infection with each of the four PTLV groups, control *tax* templates were generated from PTLV-infected humans, NHPs, or cell lines with primers PH2FN and AV43 and cloned into pCR2.1 TOPO (Invitrogen, Carlsbad, CA).

BLAST analysis of individual sequences was performed at the National Center for Biotechnology Information website using the default parameters (http://blast.ncbi.nlm.nih.gov/) to identify highly related sequences for comparison. SFV *pol* and PTLV LTR sequences were aligned using MAFFT [38] with reference sequences available at GenBank and new sequences representative of a number of NHP species, including some endemic to the Côte d’Ivoire. Unreliable columns in the alignment below a confidence score of 0.93 were then identified and removed using the program Guidance2 [39] available at http://guidance.tau.ac.il/ver2/. Phylogenies were constructed using Bayesian inference as described previously [11, 14, 40] and nucleotide substitution models were inferred using the maximum likelihood method with goodness of fit measured by the Bayesian information criterion in MEGA v6.0.6 [41]. The general time reversible model with Gamma distributed rates (Γ) and proportion of invariable sites (I) and the Hasegawa-Kishino-Yano (HKY) + Γ was inferred as the best nucleotide substitution model available in BEAST for the SFV *pol*, and PTLV *tax* and HTLV-2 LTR alignments, respectively. Bayesian trees were inferred using the program BEAST v1.8.2 and the appropriate nucleotide substitution model with an uncorrelated lognormal relaxed-clock model of nucleotide substitution and a birth-death speciation process tree prior and 100–150 million Markov chain Monte Carlo (MCMC) chains [42]. Convergence of the chain sampling was checked in the program Tracer for effective sample sizes (ESS) > 150. Trees were saved every 10,000–15,000 generations and the tree with the maximum product of the posterior clade probabilities (maximum clade credibility tree) was chosen from the posterior distribution of 10,001 sampled trees after burning in the first 1,000 sampled trees with the program TreeAnnotator version 1.8.2. Trees were viewed in FigTree version 1.4.1 (http://tree.bio.ed.ac.uk/software/figtree/).

### HIV/SIV PCR and sequence analysis

RNA was extracted from 200 μL plasma using a Qiagen UltraSens RNA kit and reconstituted in 50 μL of assay buffer. HIV-1 protease (*pro*)-*pol* sequences were amplified from 5 μL RNA by reverse transcriptase-PCR (RT-PCR) using the primary reverse and forward primers RTP-REV2 5′-CTT CTG TAT GTC ATT GAC AGT CC-3’ and RTP-F1 5′-CCT CAG ATC ACT CTT TGG CAA CG-3’ and the nested PCR primers HIV PR/RT.2F 5’ CAG GTC ACT CTT TGG CAA CGA CC-3’ and HIV-RT 181~190.1.4R 5’-ATC AGG ATG GAG TTC ATA ACC CA-3’, respectively, for 40 cycles for each round of PCR of 95°C for 45 sec, 50°C for 30 sec, and 72°C for 2 min [43]. Additional nested oligoprimers used with the above conditions included the primary HIV-B RT Gen.2F 5’-AAA GTT AAA CAA TGG CCA TTG ACA G-3’ AND HIV-B RT Gen.4R 5’-ATC CCT GCA TAA ATC TGA CTT GC-3’ and nested HIV-RT 215–219.3R 5’-CTT CTG TAT GTC ATT GAC AGT CC-3’ and HIV-RT41~69 Seq 1F 5’-TGG CCA TTG ACA GAA GAA AAA ATA AAA GC-3’ primers [43]. HIV-1 viral loads were not performed due to limited volumes of the archived plasma specimens.

DNA lysates were prepared for persons with archived PBMCs and preliminary HIV-2 and HIV undifferentiated Multispot serologic results. DNA integrity was evaluated by ß-actin PCR as previously described [ref]. Generic HIV-2 PCR primers were used to test these DNA specimens for the presence of HIV-2 *nef*/LTR sequences using outer primers LTRA and LTRB and inner primers LTRC and LTRD and conditions reported in detail elsewhere [44]. In addition, ~ 600-bp *pol*-integrase sequences were PCR-amplified from the PBMC DNA using the first and second round generic primers POLIS4 and POLOR and POLIS2 and UNIPOL2, respectively, and conditions described elsewhere [45].

HIV genotype was inferred using the internet-based COntext-based Modeling for Expeditious Typing version 0.2 (COMET HIV-1/2; https://comet.lih.lu/) and Geno2pheno (http://www.geno2pheno.org/) tools.

### GenBank accession numbers

The new SFV *pol* and LTR sequences generated in the current study have been deposited in GenBank with the accession numbers KU680639-KU680665 and KU682278, respectively. The new AGM COX2, and HTLV LTR and tax sequences have the accession numbers KU680574-KU680618, KU680619-680627, KU680628-KU680638, and the new HIV *pol* sequences have the accession numbers KU680666-KU680673, respectively. GenBank does not accept sequences < 200 nucleotides in length and thus the HIV-2 LTR sequences (~139 nt) do not have accession numbers but rather are provided in the Supporting Information (S1 File).

## Results and Discussion

### Single and dual retrovirus infections in Côte d’Ivoire

Archived plasma, some with matching peripheral blood mononuclear cells (PBMCs), were available from 1,539 hospitalized patients, pregnant women and newly diagnosed tuberculosis (TB) patients in various hospitals in Abidjan, Côte d’Ivoire consenting to participate in different studies. Specimens were collected in 1985–1988 (n = 452), 1987–1988 (n = 670) and 1995–1996 (n = 417) as part of different cross-sectional HIV studies at Project RETRO-CI [23–27, 46] (Table 1). Additional demographic, clinical, and epidemiological information for these specimens is no longer available. A subset of plasma samples (n = 858) were randomly selected for HIV testing using the BioRad GS HIV-1/HIV-2 *PLUS O* EIA based on available specimen volume to confirm the previously reported prevalence of HIV-1 and HIV-2 using antibody-based commercial assays. 428/858 (50%) plasma samples were HIV EIA reactive which is within the 46–71% infection rate range previously reported for TB and hospitalized patients in Côte d’Ivoire during this period [23–27, 46]. Enough plasma remained from 390/428 (91%) specimens for testing by the Multispot HIV-1/HIV-2 Rapid test to estimate the prevalence of HIV-1 and/or HIV-2. 307/390 (79%) were positive for HIV-1 antibodies, 8/390 (2%) were positive for HIV-2 antibodies, 17/390 (4%) were positive for HIV antibodies but could not be differentiated as HIV-1 or HIV-2 and may represent dual HIV or SIV infections, while 83/390 (21%) samples were negative. Examination of these results by study period showed that 105/127 (84%) from 1985–88, 42/67 (63%) from 1987–88, and 186/196 (95%) collected during 1995–96 were Multispot-positive (Table 1). Six of eight (75%) HIV-2 samples were from 1987–88 and two (25%) were from 1995–96. One of 17 (6%) undifferentiated samples was from 1985–88, 9/17 (53%) from 1987–88, and 8/17 (47%) were from 1995–96. These results are all comparable to those previously reported for this population indicating the viability of the archived samples for testing for other retroviruses [23–27, 46].

**Table 1:** Detection of simian and human retrovirus infections in Côte d’Ivoire 1985–1997^1^^,^^2^.

Collection period	N	SFV EIA pos	SFV WB pos	SFV PCR pos	HTLV EIA pos	HTLV WB reactive^3^	HTLV PCR pos^3^	HIV EIA pos^4^	HIV Multispot pos^3^	HIV/SIV PCR pos^3^
***1985–88***	452	17 (3.8)	2 (0.4)	ND^5^	26 (5.7)	27 (5.1); 21 HTLV-1, 1 HTLV-2, 1 untypeable^6^, 4 IND^7^	ND	129/441 (29.3)	105/127 (83.5); 104 HIV-1, 1 HIV undifferentiated	ND
***1987–88***	670	8 (1.2)	0	ND	26 (3.9)	26 (2.6); 14 HTLV-1, 2 HTLV-2, 2 untypeable, 8 IND	ND	80/130 (61.5)	42/67 (62.7); 27 HIV-1, 6 HIV-2, 9 HIV undifferentiated	ND
***1995–96***	417	1 (0.2)	1 (0.2)	1 (SFVcsa)^8^	18 (4.3)	18 (3.4); 11 HTLV-1, 1 HTLV-2, 2 untypeable, 4 IND	10/15 (66.7); 7 HTLV-1, 2 STLV-1-like, 1 HTLV-2	219/287 (76.3)	186/196 (94.8); 176 HIV-1, 2 HIV-2, 8 undifferentiated	7/8 (87.5); 3 HIV-2, 3 dual HIV-1/HIV-2, 1 HIV-1
**Totals**	**1,539**	**26 (1.7)**	**3 (0.2)**	**1 (0.07)**	**71 (4.6)**	**71 (4.6)**	**10/15 (66.7)**	**428/858 (49.9)**	**336/390 (86.1)**	**7/8 (87.5)**

^1^. pos = positive, percentages in parentheses

^2^. Serotypes and genotypes given when determined per confirmatory serologic assay or by PCR testing and phylogenetic analysis

^3^. Prevalences for each time period determined only for WB- or PCR-positive specimens and specimen availability; summary of WB profiles or retrovirus subtypes given after prevalences

^4^. Only a subset of plasma samples (n = 858) were tested for HIV

^5^. ND, archived specimens not available for testing

^6^. HTLV-positive but not typeable as genotypes 1 or 2.

^7^. IND, indeterminate test results

^8^. SFVcsa originates from *Chlorocebus sabaeus* (csa)

**Fig 1:**
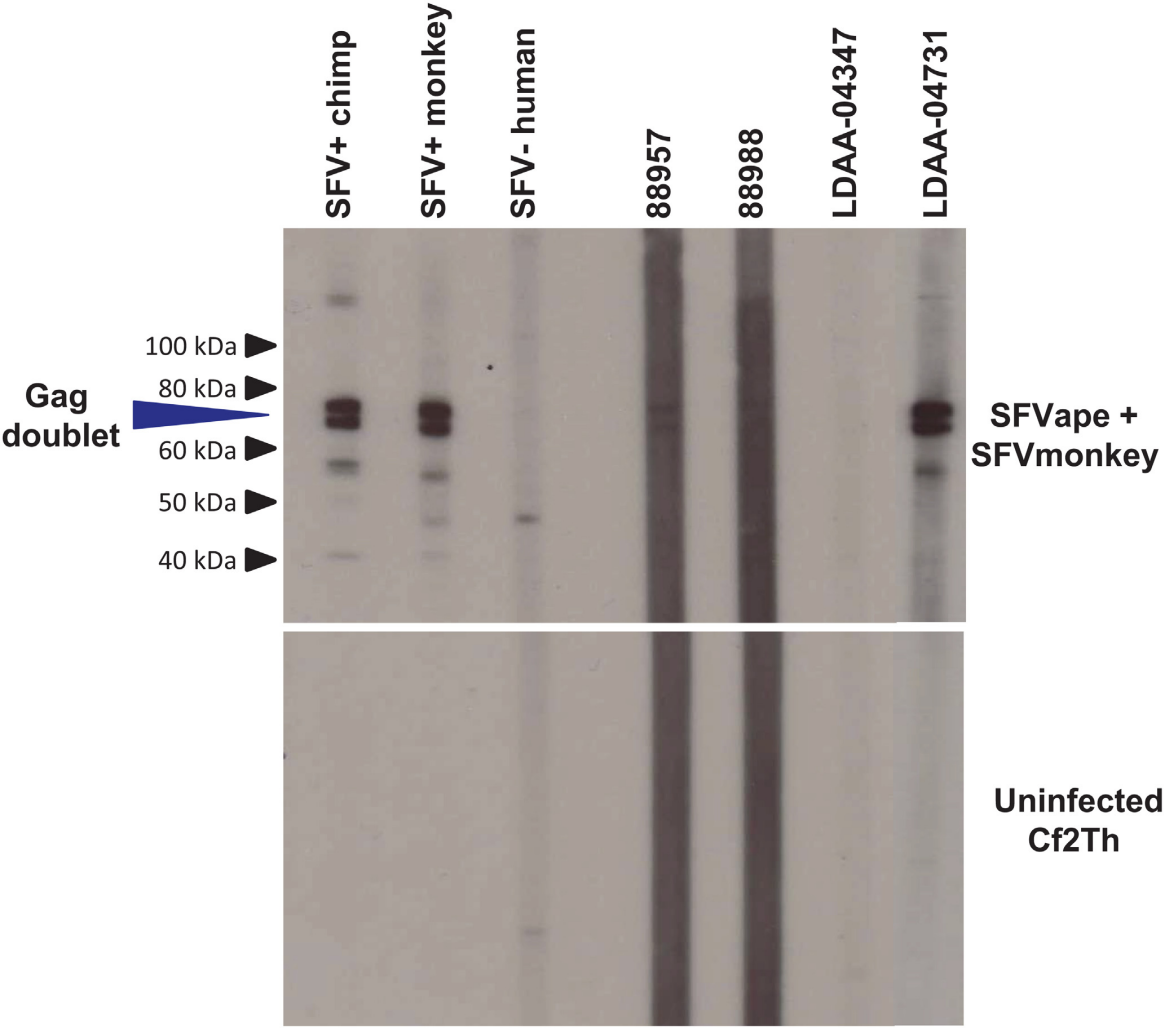
Western blot detection of antibodies to simian foamy virus (SFV) in persons from Côte d’Ivoire, 1985–1996. Upper panel shows seroreactivity of representative patient samples to the combined SFV antigens from ape (chimpanzee) and African green monkey infected cell cultures. Lower panel shows reactivity to crude cell lysate antigens from uninfected canine thymocytes (Cf2Th). Seroreactivity was defined for those specimens with reactivity specific to the diagnostic Gag doublet proteins (p72/p74) in the combined antigen assay. Patient LDAA-04731 is co-infected with HIV-1.

26/1,539 (2%) plasma specimens were reactive in the SFV EIA of which three (12%) tested Western blot (WB) seropositive, showing reactivity to the diagnostic SFV Gag doublet proteins, giving an overall prevalence of 0.2% (3/1,539), 95% confidence limits (CL) (0.05–0.6) (Table 1). The WB assay has been previously shown to be 100% specific and sensitive for confirming infection with variant SFV [31]. Fig 1 displays the SFV WB reactivity of both seropositive (88957, 88958, and LDAA-04731) and seronegative (LDAA-04347) specimens. Both 88957 and 88958 specimens were collected in 1985–88 while LDAA-04731 was collected during 1995–96. The older specimens 88957 and 88958 gave weaker anti-SFV responses than LDAA-04731. The SFV prevalence seen in Côte d’Ivoire is comparable to that we reported earlier in DRC (0.5%) and Cameroon (1%) but contrasts with the 11%– 19% infection rate seen in Cameroon and Gabon in highly exposed subgroups who sustained severe bites and wounds from NHPs [9, 10, 36, 47]. Thus, the lower rate in our study likely better represents infection prevalence in the general population compared to the higher rate seen in highly exposed persons in previous studies. While patients in these previous studies were from hospitals or had specific infections, their health information was not stored with the specimens to evaluate SFV infection with disease status though the prevalence was likely too low for such inferences.

71/1,539 (4.6%) plasma specimens were repeatedly HTLV EIA reactive and all were also WB reactive (Table 1); 47 were HTLV-1-like (66.2%), four were HTLV-2-like (5.6%), five (7.0%) were HTLV-positive but untypeable as either HTLV-1 or HTLV-2, and 16 (22.5%) were seroindeterminate. The overall HTLV WB-positive prevalence was 3.6% (56/1,539). During the period of 1985–1987 the WB prevalence was 3.9% compared to 3.4% during 1995–1996 suggesting a relatively stable infection rate over ten years. We found a higher prevalence of HTLV antibodies than that reported recently in persons from rural Côte d’Ivoire (1.3% 16/776), and a similar prevalence as that reported from studies of mixed populations from the same decade as our two earliest collections, which ranged from 1.5–4.5% [48–52]. When compared to subpopulations in Côte d’Ivoire in these and other studies, patients with AIDS, leprosy and HAM/TSP had higher HTLV seroprevalences of 7.2%, 9.9%, and 15%, respectively, than those found in our population of hospitalized patients [49–52] which may reflect differences in disease severity between the various studies.

Two of the three SFV WB-positive persons (88957 and LDAA-04731) were also HIV-1-positive by the Multispot test but all three were negative for antibodies to HTLV. None of the four HTLV-2-positive or the five HTLV-positive untypeable samples were also positive for HIV. One of the eight (12.5%) HIV-2-positive samples and 1/18 (5.6%) undifferentiated HIV samples were HTLV-1-positive and both were collected during 1987–88. In contrast, 14 persons were dually infected with HTLV-1 and HIV-1; 3/14 (21.4%) from 1987–88, 8/14 (57.1%) from 1985–88, and 3/14 (21.4%) from 1995–96. Four of 12 (33.3%) samples with HTLV WB indeterminate results were also positive for HIV-1 antibodies, one each from 1985–88 and 1987–88, and two from 1995–96.

This is the first report of SFV infection in both West Africa and specifically in Côte d’Ivoire and expands further the range of SFV-infected humans in Africa. Our finding of dual HIV and SFV infections is the second such report; our initial finding was an HIV-1-infected blood donor in Cameroon who was dually infected with SFV from a mandrill, a monkey species endemic in Cameroon [53]. Our finding of SFV infection in specimens collected from 1985–1996 suggests that SFV likely crossed into humans in this region decades ago and could continue to spread cryptically because the conditions and the associated exposures for these infections have not changed and since blood bank screening is not mandated or conducted for SFV [54]. While these results may not be surprising given the history of NHP hunting in Côte d’Ivoire, they reiterate the importance of defining the disease potential of SFV in humans. Defining SFV pathogenicity in humans has been difficult because all SFV-infected persons have to date have been identified in surveys of predominantly healthy populations which helped document SFV emergence but precluded the identification of disease associations. However, our ability to detect dual HIV-1/SFV infections in this study may provide an alternative approach to study SFV pathogenicity in the setting of HIV-related immunosuppression, and support expanding these surveys to identify larger numbers of co-infected individuals to permit assessment of SFV-associated disease.

### PCR and phylogenetic analysis of new SFV and COX2 sequences

**Fig 2:**
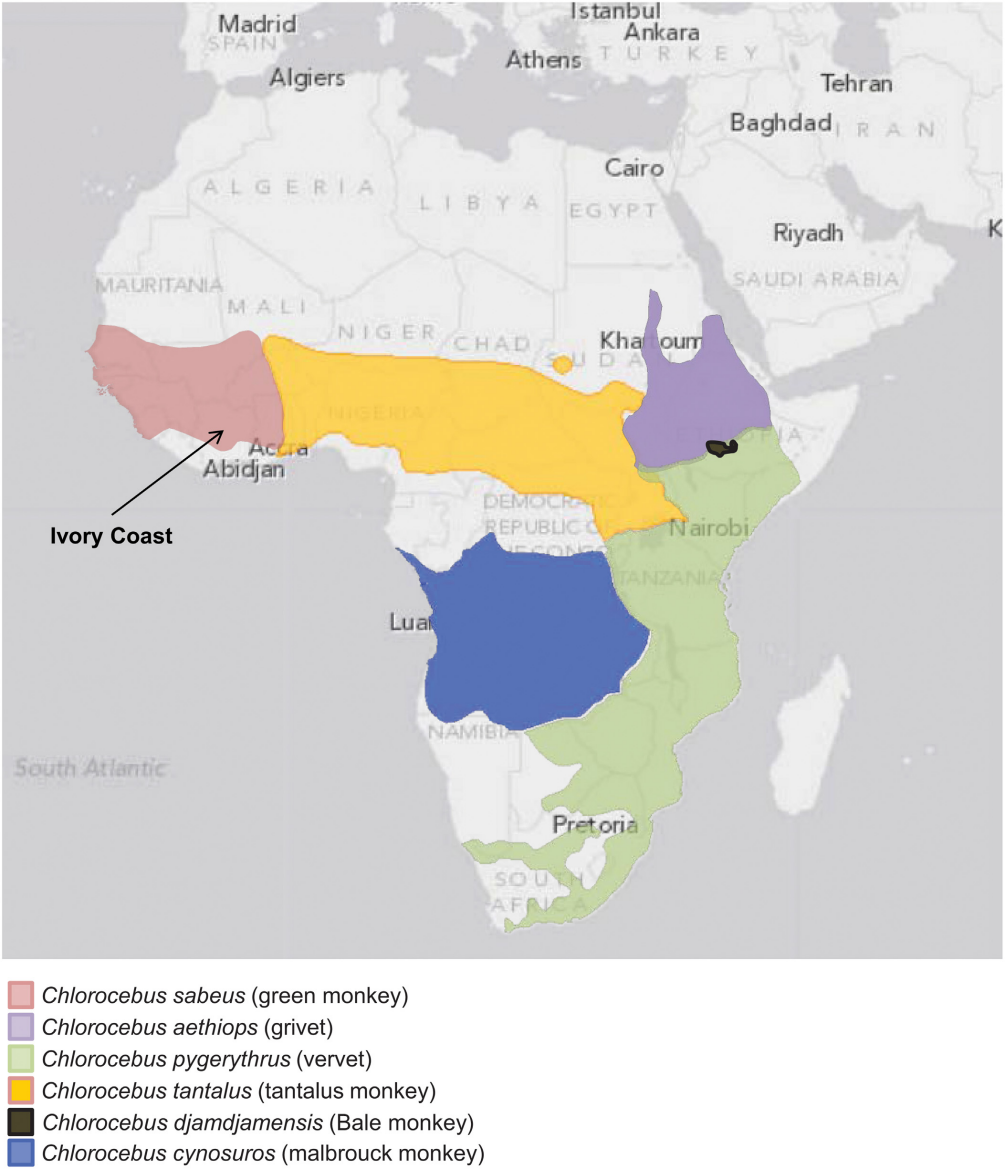
Distribution of African green monkeys (*Chlorocebus* species) in Africa. *Chlorocebus* classification and ranges are from the International Union for Conservation (IUCN) of Nature Red List (http://www.iucnredlist.org/search) downloaded Nov. 18, 2015.

**Fig 3:**
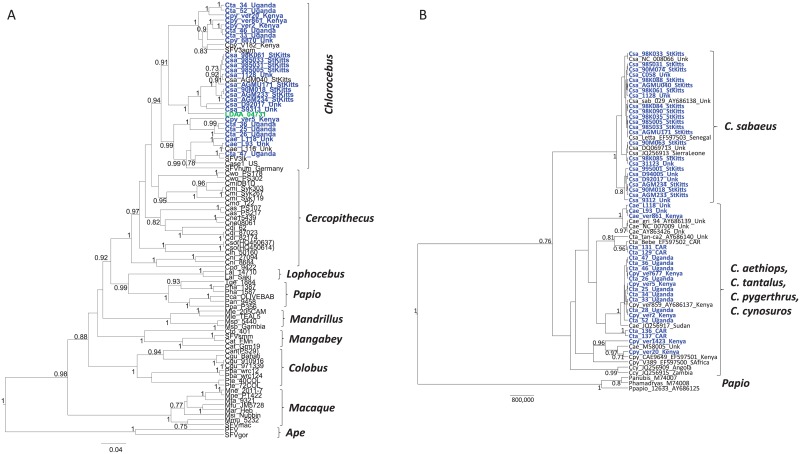
Inference of the evolutionary history of human infection with simian foamy virus (SFV). **A**. Bayesian SFV polymerase (*pol)* maximum clade credibility tree inferred using an alignment of 375-bp and 88 Old World monkey and ape taxa, including 26 new African green monkey sequences (in blue text). The SFV *pol* sequence from the Côte d’Ivoire patient, LDAA-04731, is shown in green text. **B**. *Chlorocebus* mitochondrial DNA phylogenetic relationships inferred by Bayesian analysis of an alignment of 496-bp from 65 taxa. New sequences generated in our study are highlighted in blue text. Animal country of origin given at end of taxa names; CAR = Central African Republic, Unk = provenance unknown. Csa, *Chl*. *sabaeus*, Cae, *Chl*. *aethiops*, Cta, *Chl*. *tantalus*, Cpy, *Chl*. *pygerythrus*, Ccy, *Chl*. *cynosuros*. Posterior probabilities > 0.7 are given at major nodes in the tree. Scale bar is in units of time.

Archived PBMCs were available from one of the three SFV-WB-positive persons for PCR analysis. LDAA-04731 was positive for SFV polymerase (*pol*) and LTR sequences using nested PCR. A BLAST search of the LDAA-04371 SFV *pol* sequences showed about 92% nucleotide identity to those from African green monkeys (AGM). Six species of AGM are currently recognized with wide geographic spread across Africa, including *Chlorocebus sabaeus* (green monkey), *Chl*. *aethiops* (grivet), *Chl*. *pygerythrus* (vervet), *Chl*. *tantalus* (tantalus monkey), *Chl*. *djamdjamensis* (Bale monkey) and *Chl*. *cynosures* (malbrouck monkey) (Fig 2) [55]. However, only one or two SFV *pol* sequences are available from three AGM species (*Chl*. *sabaeus*, *Chl*. *aethiops*, *Chl*. *pygerythrus*), with others reported as AGM when the species or provenance was unknown, including those from two other SFV-infected humans [14, 56]. Thus, to more precisely determine the primate origin of SFV infection in person LDAA-04371, we PCR-amplified SFV *pol* sequences from 26 additional AGMs from four species, including eight tantalus wild caught monkeys from Uganda, four wild-caught vervets from Kenya, 11 green monkeys from the Caribbean island of St. Kitts, and three grivets bred in captivity but for which the country of origin was not provided. Phylogenetic relationships of 375-bp SFV *pol* sequences from 88 infected NHPs and humans were inferred by Bayesian methods and showed an overall co-evolution of host and virus with strong statistical support (Fig 3a). The LDAA-04731 *pol* sequence clustered with very high posterior probability ((PP) = 1) within the diversity of the monophyletic *Chl*. *sabaeus* (Csa) SFV clade. In contrast, *pol* sequences from two other previously reported SFVagm-infected persons (Case 1 and SFVhum) clustered within a mixed AGM clade containing sequences from grivets (Cae), vervets (Cpy), and tantalus (Cta) monkeys with strong support (PP = 0.99) (Fig 3). Of the four AGM species, only SFV from *Chl*. *sabaeus* was monophyletic which is unusual given the co-evolutionary history of SFV. Thus, to confirm the AGM species of these specimens, we performed neighbor joining analysis of 45 new and 20 reference mitochondrial cytochrome oxidase subunit II (COX2) sequences. These results also showed a monophyletic cluster of *Chl*. *sabaeus* (Csa) sharing a common ancestor with paraphyletic COX2 sequences from *Chl*. *tantalus* (Cta), *Chl*. *aethiops* (Cae), and *Chl*. *pygerythrus* (Cpy) (Fig 3b). Others have reported the same general phylogenetic relationships using mtDNA cytB sequences and suggested the results are likely from hybridization occurring in past and present overlapping habitats of the latter three AGM species (Fig 2) [55]. Specimens were not available from Bale and malbrouck monkeys for analysis herein but for which phylogenetic analysis of CytB sequences in an earlier study showed that they did not cluster with *Chl*. *sabaeus* and thus would likely not have influenced our results [55]. Our findings, combined with those from other studies, show that simian retroviruses from a variety of NHPs are crossing into humans in this region and not from only sooty mangabey monkeys as proposed by Calvignac-Spencer et al. [22].

Phylogenetic analysis of the LTR sequences was not performed since only a small number of SFV sequences from a limited number of simian species are available at GenBank for comparison. In addition, the LTR region is highly divergent and thus alignments with all available SFVs do not contain enough phylogenetic signal to accurately resolve the genetic relationships [12]. By BLAST analysis, the LTR sequence from person LDAA_04731 shared about 90% nucleotide identity with LTR sequences from African green monkey (SFV-3, M74895), spot-nosed guenon (AG16, JQ867466 and CAM2467LE, AY390392), and red-tailed guenon (40224, JX157540).

### PCR and phylogenetic analysis of new HTLV sequences

**Fig 4:**
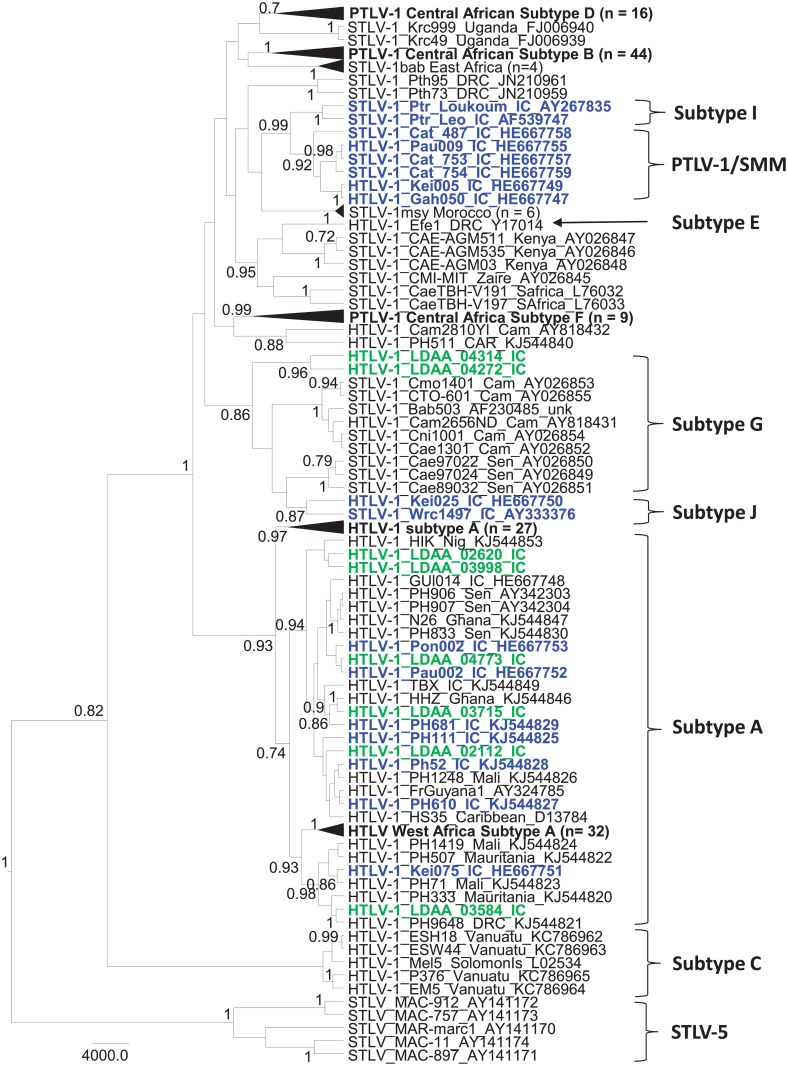
Identification of simian T-lymphotropic virus type 1 (STLV-1)-like viruses in patients from Côte d’Ivoire. Bayesian long terminal repeat (LTR) maximum clade credibility tree inferred using an alignment of 1,178 nucleotide positions and 216 global STLV-1 and human T-lymphotropic virus type 1 (HTLV-1) taxa. New LTR sequences created in our study are shown in green text, while those from other studies from Côte d’Ivoire are shown in blue text for comparison. Taxa in some subtypes are collapsed for clarity with the number of taxa given in parentheses. Posterior probabilities > 0.7 are given at major nodes in the tree. Scale bar is in units of time.

Archived PBMCs for PCR testing were available for 15/71 (21%) persons with reactive HTLV-1 WB results, including HTLV-1 (n = 9), HTLV-2 (n = 1), and HTLV indeterminate (n = 5) seroreactivity. Using generic PTLV-1 PCR primers we obtained *tax* sequences from 11 persons (73.3%). BLAST analysis showed that 10/11 (90.1%) *tax* sequences were HTLV-1 and one (9.1%) was HTLV-2. Eight of the ten HTLV-1-infected persons had HTLV-1 WB profiles, two had indeterminate WB results while the HTLV-2-infected person had an HTLV-2 WB profile (data not shown). To further investigate the genetic relationships of HTLV in these persons we analyzed LTR sequences, which is one of the more divergent PTLV genomic regions used to differentiate subtypes within both HTLV-1 and HTLV-2 [29, 32]. We successfully obtained 8 HTLV-1 and one HTLV-2 LTR sequences, nearly matching the *tax* results. We were unable to amplify LTR sequences from two persons (LDAA_04445 and LDAA_02481) with indeterminate and HTLV-1 WB profiles, respectively. Phylogenetic analysis of LTR sequences showed that PTLV-1 from two persons (LDAA_04314 and LDAA_04272) clustered strongly together (PP = 0.96) and within the subtype G clade (PP = 0.86) which contains STLV-1 from a variety of NHP species, including *Chl*. *aethiops* from Senegal and one possible zoonotic HTLV-1 infection from a primate hunter in Cameroon (Cam2656ND) (Fig 4). This clustering suggests possible cross-species transmission of STLV-1 to these two persons from Côte d’Ivoire (Fig 4). In addition, the tight clustering of these two sequences suggests a possible epidemiological linkage of these infections. Nonetheless, a specific primate species could not be directly linked to their infections as STLV-1 sequences were paraphyletic to these two Côte d’Ivoire HTLV-1 sequences in this monophyletic clade. Subtype J sequences also clustered strongly with the subtype G sequences in this analysis (Fig 4). The subtype J clade was named by Calvignac-Spencer et al. [22, 57] and in their recent study included one HTLV-1 sequence from a person (Kei025) from Côte d’Ivoire who reported dismembering and eating monkeys, including several STLV-1 from western colobus monkeys (*Piliocolobus badius badius*) and chimpanzees (*Pan troglodytes verus*) [22]. Similarly, their classification of subtype I STLV-1 from other *P*. *t*. *verus* and *P*. *b*. *badius* from Côte d’Ivoire that cluster strongly in a monophyletic clade with STLV-1 from sooty mangabeys (*Cercocebus atys*) and HTLV-1 from Côte d’Ivoire [22, 57] agree with our analyses (Fig 4). However, their classification of these STLV-1 as two separate subtypes is not clear and perhaps should be collapsed into a single subtype. Further analysis of additional gene regions or complete genomes may be needed to determine if the G and J and I and STLV-1sm clades are truly monophyletic as inferred in our phylogenetic analysis.

**Fig 5:**
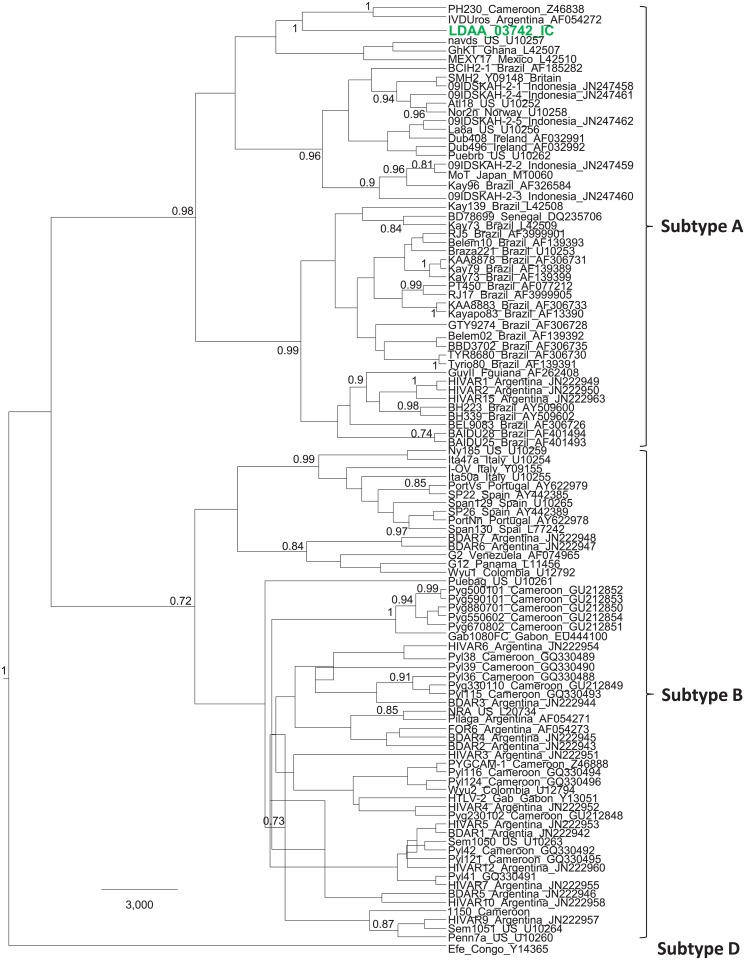
Evolutionary history of human T-lymphotropic virus type 2 (HTLV-2) in Côte d’Ivoire. Bayesian long terminal repeat (LTR) maximum clade credibility tree inferred using an alignment of 692 nucleotide positions and 105 global HTLV-2 taxa. The new Côte d’Ivoire HTLV-2 LTR sequence created in our study is shown in green text. Posterior probabilities > 0.7 are given at major nodes in the tree. Some subtype B taxa were inferred to have negative branch lengths indicative of a descendant node older than its direct ancestor, and is not present in the ancestral clade trees of the MCC tree. Scale bar is in units of time.

The remaining six LTR sequences from persons from Côte d’Ivoire in our study clustered within the cosmopolitan subtype A clade, and in most cases with those from West Africa, including those found in Côte d’Ivoire (Fig 4). The HTLV-2 LTR sequence clustered strongly (PP = 1) within the subtype A clade with a sequence from Cameroon (PH230) and one from Argentina (Fig 5). Although HTLV-1 has been previously identified in Côte d’Ivoire using serological tools, to the best of our knowledge this is the first HTLV-2 infection confirmed from this country and only the 3rd HTLV-2 subtype A strain reported from Africa (strains PH230 from Cameroon and GhKT from Ghana). Earlier studies conducted in the late 1980s and early 1990’s used mostly EIAs that could not differentiate HTLV-1 from HTLV-2 and sequence analysis was not performed for genotyping [49–51]. Only one case of possible HTLV-2 infection was reported using only type-specific serological tools in those previous studies [48]. Thus, our confirmation of HTLV-2-infection in Côte d’Ivoire is significant and shows a broader distribution of this deltaretrovirus across Africa. Aside from the recent study by Calvignac-Spencer et al. [22], ours is the only other study to use molecular sequences to investigate the genetic diversity of HTLV in Côte d’Ivoire. While HTLV-1 causes disease in only about 5% of infected persons, little is known about the health consequences of STLV-1-like infection since these persons have not been systematically studied and retroviral disease can take decades to present [22, 32]. However, one recent study identified a chronic progressive neurological disease, HTLV-associated myelopathy/tropical spastic paraparesis (HAM/TSP), in a patient from Liberia. This person had an STLV-1-like infection most similar to STLV-1 from sooty mangabeys in Côte d’Ivoire similar to those found by Calvignac-Spencer et al. These findings support the disease potential of such zoonotic STLV infections [22, 58].

### HIV sequence analysis

Enough plasma specimen was remaining from two of the SFV-reactive HIV-1 co-infected persons (88957 and LDAA_04731) to allow HIV-1 subtyping using *pol* sequences. The 88957 and LDAA_04731 *pol* sequences were determined to be subtypes B and circulating recombinant form CRF02_AG, respectively, using two automated subtyping tools (COMET and Geno2pheno) (Table 2). Subtype CRF02_AG has been circulating in Côte d’Ivoire since the time specimens in our study were collected, and is the predominant strain in Côte d’Ivoire. However, subtype B is not very common in West Africa, including Côte d’Ivoire [59]. BLAST analysis of the 88957 *pol* sequence showed it had about 97% nucleotide identity to other global subtype B sequences, including Kenya (GenBank accession number KP8779473) and South Africa (JN638211).

**Table 2:** Detection of HIV dual infections in Côte d’Ivoire 1985–1997.

Specimen Code	Collection Period	Specimen type	HIV Multispot Results	*pol* genotype^1^	HIV-2 LTR genotype	B-actin PCR results^2^
88957	1985–1988	plasma	Preliminary Positive for HIV-1 Antibodies	HIV-1 B	ND	ND
763094	1995–1996	PBMCs^3^	Preliminary Positive for Antibodies to HIV Undifferentiated^4^	HIV-2 A	A	Pos
763095	1995–1996	PBMCs	Preliminary Positive for Antibodies to HIV Undifferentiated	HIV-1 CRF02_AG	B	Pos
763127	1995–1996	PBMCs	Preliminary Positive for HIV-2 Antibodies	Neg	B	Pos
763128	1995–1996	PBMCs	Preliminary Positive for Antibodies to HIV Undifferentiated	ND	ND	Neg
763137	1995–1996	PBMCs	Preliminary Positive for Antibodies to HIV Undifferentiated	HIV-1 CRF02_AG	B	Pos
LDAA-04731	1995–1996	plasma	Preliminary Positive for HIV-1 Antibodies	HIV-1 CRF02_AG	ND	ND
LDAA-04829	1995–1996	PBMCs	Preliminary Positive for Antibodies to HIV Undifferentiated	HIV-1 CRF02_AG	Neg	Pos
LDAT-02091	1995–1996	PBMCs	Preliminary Positive for Antibodies to HIV Undifferentiated	HIV-1 A1	A	Pos
LDAA-03666	1995–1996	PBMCs	Preliminary Positive for Antibodies to HIV Undifferentiated	HIV-2 A	A	Pos

^1^. PCR or RT-PCR using generic HIV/SIV or HIV primers, respectively, as described in the methods

^2^. ND, not done; Pos, positive; Neg, negative

^3^. PBMCs, peripheral blood mononuclear cells

^4^. Undifferentiated, could not differentiate from HIV-1 and HIV-2

Archived PBMC, but not sufficient plasma volumes, were available from 8/26 (30.7%) persons with HIV EIA reactivity who were preliminary positive for HIV-2 antibodies (n = 1) or with undifferentiated HIV results (n = 7) in the Multispot assay. Given our evidence of both SFV and STLV-1-like infections in this population, we tested the PBMC DNA from these persons for SIV using degenerate *pol* and LTR PCR primers capable of detecting diverse SIVs [44, 45]. HIV-2 LTR sequences were detected in 6/7 (85.7%) persons but SIV sequences were not detected in these specimens (Table 2). There was an equal distribution of HIV-2 subtype A and subtype B in these six PCR-positive samples. Again, 6/7 (85.7%) persons were also positive for *pol* sequences, none of which were SIV-like. Of these six, one (763127) was positive for HIV-2 subtype B LTR sequences but negative for *pol* sequences and one specimen (LDAA-04829) negative for HIV-2 LTR sequences was positive for HIV-1 CRF02_AG *pol* sequences. Of the remaining five *pol*-positive samples, two (763094 and LDAA-03666) were positive for HIV-2 subtype A, two (763095 and 763137) for HIV-1 CRF02_AG, and one with HIV-1 subtype A1 *pol* sequences (LDAT-02091). Three HIV-1 *pol*-positive samples (763095, 763137, and LDAT-02091) were dually infected with HIV-2 by LTR testing. The two HIV-1 CRF02_AG infections were dually infected with HIV-2 subtype B, while the HIV-1 A1-infected person was co-infected with HIV-2 subtype A. One specimen tested negative in both PCR tests and was also negative for ß-actin sequences suggesting poor DNA quality of the sample.

HIV-1 subtypes CRF02_AG and A1 predominate in Côte d’Ivoire (about 66% and 20%, respectively), whereas HIV-2 subtype B is slightly more frequent than subtype A (44% vs 36%), both of which match our results from testing of small numbers of samples and when combined with HIV seroprevalence results confirms the representativeness of the subpopulation sampled for HIV testing herein. While dual HIV infections were common in Côte d’Ivoire during the period our study was conducted, and remain common today, little information is available on the genotypes present in dual infections. Thus, our results may provide important baseline information for future studies examining the clinical effects of dual infection or for studies involving the improved detection and discrimination of HIV types in co-infected persons.

Though both HIV-1 and HIV-2 originated from cross-species SIV infections, with HIV-2 likely originating from SIVsmm from an infected sooty mangabey monkey (smm), we did not find any evidence of SIV-like infections in our population. Sequence analysis of all HIV-positive samples in our study would likely improve the opportunity to identify such infections but specimens for that analysis were not available. Nonetheless, anonymous testing of specimens from Côte d’Ivoire collected in 2006–2007 has recently demonstrated that a divergent SIVsmm crossed into an 8-year old child who reportedly consumed primate bushmeat, suggesting that zoonotic SIV infections continue to emerge in this region of Africa [21]

### Proviral loads (pVL) in SFV- and HTLV-infected persons

HIV-1 viral loads were not determined for the persons from Côte d’Ivoire dually infected with SFV or PTLV because of limited plasma volumes. The SFV PBMC pVL for person LDAA_04731 was determined to be 123 copies/ug DNA using a recently described qPCR assay [12]. This viral load is within the range reported for infected humans and NHPs suggesting that dual infection with HIV-1 in this person may not have affected their SFV pVL [12, 60, 61]. For comparison, we also measured pVLs in the blood donor from Cameroon (BB9) who we previously reported was dually infected with HIV-1 and SFV from a mandrill [53]. The PBMC DNA for BB9 showed an SFV pVL of 17.8 copies/ug and the plasma HIV-1 viral load was 5,800 copies/ml from previous testing [62]. While SFV and SIV/HIV interactions remain poorly understood in vivo, some impact of SFV on SIV has been reported in macaques that were naturally infected with SFV and received experimental SIV infection [63]. Compared to SFV-negative macaques, these animals had both increased SIV viremia and SIV-associated disease progression [63].*In vitro* SFV-infected cells increase the ability of HIV-1 binding gp120 envelope proteins to cell surface heparin-sulfate proteoglycans [64]. Thus, clinical and virologic follow-up of SFV/HIV-1 in co-infected persons may be needed to assess risks of accelerated HIV-1 disease and the need for early initiation of antiretroviral therapy.

For HTLV, higher viral loads in both HTLV-1 and -2-infected persons have historically been shown to be associated with increased pathogenicity, including development of leukemia/lymphoma and HAM/TSP, and also increased transmissibility [65–71]. However, the majority of qPCR assays used to date were designed to measure viral loads for only HTLV-1 or HTLV-2 individually. More recently, two multiplex assays have been described that utilize combinations of PTLV-1, PTLV-2, and PTLV-3-specific primers and probes but none have been developed to also detect PTLV-4 [72, 73]. However, multiplex qPCRs can have lowered detection sensitivities due to saturation of the reaction with higher levels of the primer/probe mixtures. Thus, we designed a new qPCR assay that uses a single set of generic primers and probes in the highly conserved *tax* gene to detect and determine proviral loads of PTLV-1, PTLV-2, PTLV-3, and PTLV-4. Our new generic qPCR test has a sensitivity of detecting 10 copies of prototypical HTLV-1_ATK, HTLV-2_G12, HTLV-3_Cam2026ND, STLV-3d_Cam8699, HTLV-4_Cam1863LE, and the highly divergent STLV-1_MarB43 tax sequence against a background of 1 ug HTLV-negative blood donor DNA (equivalent to ~125,000 cells). In addition, 100 HTLV-negative blood donor DNA specimens tested negative in this test. The average proviral load in the HTLV-1-infected persons from Côte d’Ivoire was 2.44E+04/ug DNA (19.5 copies/100 cells) and ranged from < 10 copies (below the assay detection threshold) to 1.28E+05/ug DNA (102.4 copies/100 cells). The highest pVLs, 1.28E+05/ug DNA (102.4 copies/100 cells) and 1.13E+04/ug (9.1 copies/100 cells), were seen in two patients with subtype A HTLV-1 (LDAA-02112 and LDAA-03584, respectively). The HTLV-2-infected person (LDAA_03742) had a proviral load of 4.94E+03/ug DNA (3.9 copies/100 cells). Only the HTLV-1-infected patient LDAA-02112 had pVLs similar to those reported in patients with HTLV-related diseases (HAM/TSP and adult T-cell leukemia/lymphoma (ATLL); mean > 25 copies/100 cells) suggesting potential HTLV disease in this person but for whom clinical information was not available to confirm this finding [72, 74, 75].

## Conclusions

We document SFV and STLV-1-like infection in Côte d’Ivoire, providing evidence for expanded zoonotic simian retrovirus transmission in West Africa during the 1980s and 1990s. We also document persons with SFV/HIV-1 co-infections and identify HTLV-2 infection in Côte d’Ivoire. Phylogenetic analysis point to the specific NHP origin of SFV but data are not available to determine if these SFV infections were directly acquired from NHPs or the result of secondary spread in humans. Our results highlight the need for further studies to determine the distribution and public health consequences of SFV, STLV-1, and dual SFV/HIV infections in humans. Identification of SFV/HIV-1 co-infected populations through targeted surveys of populations with high HIV prevalence represents a new approach to study SFV pathogenicity in immunocompromised humans which may provide new insights on SFV pathogenicity and transmissibility in humans.

## Supporting Information

S1 File HIV-2 LTR sequences in fasta file format. (FAS)
